# How to Cough

**Published:** 1879-03

**Authors:** 


### HOW TO COUGH.

To some persons, coughing is harmless, but to others it is fraught with many dangers. It is, therefore, important to teach those liable to be injured by too severe or prolonged efforts at coughing how they may accomplish their purpose easily, safely and quickly. Dr. J. M. Fothergill (*Phil. Med. Times*), says: "It must be insisted upon that the chest be well filled with air before the cough is let loose; that is, the reflex act must be inhabited by the exercise of the will, until the chest be well filled with air before the cough is let loose. Such full inspiration is effective not only in removing the source of the irritation, but it usually causes other masses of mucus and charcoal to slide from their seat, and thus to set up further cough for their removal. But, if the full inspiration plan be followed, these masses are readily and quickly expelled." Of course these directions are in use only in such coughs as are for the purpose of removing some offending matter from the air passage.—*Lancet and Clinic.*

